# The Staff Nurse Clinical Leader at the Bedside: Swedish Registered Nurses' Perceptions

**DOI:** 10.1155/2016/1797014

**Published:** 2016-12-04

**Authors:** Inga E. Larsson, Monika J. M. Sahlsten

**Affiliations:** ^1^Department of Health Sciences, University West, 461 86 Trollhättan, Sweden; ^2^Department of Health and Education, University of Skövde, 541 28 Skövde, Sweden

## Abstract

Registered nurses at the bedside are accountable for and oversee completion of patient care as well as directly leading and managing the provision of safe patient care. These nurses have an informal leadership role that is not associated with any given position. Leadership is a complex and multifaceted concept and its meaning is unclear, especially in the staff nurse context. The aim was to describe registered nurses' perceptions of what it entails to be the leader at the bedside in inpatient physical care. A phenomenographic approach was employed. Interviews were performed with Swedish registered nurses (*n* = 15). Five descriptive categories were identified: demonstrating clinical knowledge, establishing a good atmosphere of collaboration, consciously structuring the work in order to ensure patients' best possible nursing care, customized presence in the practical work with patients according to predetermined prerequisites, and monitoring coworkers' professional practice. Registered nurses informal role as leader necessitates a social process of deliberate effort to attain and maintain leader status and authority. Participants used deliberate communicative approaches and interactive procedures. Leader principles grounded in the core values of the nursing profession that ensure nursing values and person-centered attributes were a key aspect.

## 1. Introduction

Registered nurses at the bedside are accountable for and oversee completion of patient care as well as directly leading and managing the provision of safe patient care [1]. These nurses have an informal leadership role that is not associated with any given position. The nurse's leader role is not prescribed or scripted; rather, it is negotiated among the actors involved and therefore subject to more or less well-articulated expectations from others. Even patients expect a registered nurse to be the leader of the nursing team that provides their care [2]. With diminishing resources, deficiencies in quality of care, and an increasing number of medical injuries, effective leadership has become essential, which is a challenge for professional nurses. Quality initiatives require leadership by front-line registered nurses [3]. Accordingly, nurses need research-based knowledge of how to implement appropriate leadership. However, leadership is a complex and multifaceted concept [4] and its meaning continues to be unclear, especially in the staff nurse context [1]. Each nurse may have his/her own experience and understanding of leadership and how to exercise it. Accordingly, nurses' perceptions of being a leader at the bedside are important knowledge for the nursing profession.

Leadership is complex and often associated with management, coaching, or mentoring, which all contribute to vagueness when it comes to implementation [5]. Research has predominantly focused on first-line managers/executives and has explored attributes, traits, competencies, roles and styles, and impact [6]. Nursing leadership has traditionally been linked with transactional leadership, not compatible with professional core values [7]. Transformational leadership has been recommended for registered nurses at the bedside [8, 9] but also Kouzes and Posner's [10] leadership, authentic leadership [11], and emotionally intelligent leadership [12, 13].

Formal leaders such as managers/head nurses gain authority by formal appointment [14]. In contrast, registered nurses at the bedside attain authority informally from acceptance and support by followers who trust in them. The maintenance of leadership is then dependent on intrateam relationships based on leaders' behaviors and followers' perceptions of these [15].

The concept of nursing leadership is used interchangeably with nursing management and literature is comprehensive and developed to support nurses with management responsibilities [5]. Research and literature in this field have been accepted as transferable and are used to acquire understanding of clinical leadership in terms of leading at the bedside. Clinical leadership is often used interchangeably and inappropriately or alongside nursing management and nursing leadership [16, 17]. Clinical leadership has been used to identify leadership qualities of staff nurses at the bedside and differentiate staff nurse clinical leadership (SNCL) from leadership in formal administrative roles [18, 19]. According to Stanley [17], clinical nurse leaders are positive clinical role models with high-level clinical competence and knowledge. They are effective communicators, remain open and approachable, visible and accessible in practice, and empowered decision-makers, and display nursing core values and beliefs through their actions. These characteristics have resulted in the theory of congruent leadership [17].

When it comes to leadership for registered nurses at the bedside or more recently staff nurse clinical leaders (SNCL), there is still a shortage of empirical studies. Research is needed in order to develop evidence-based knowledge. In this study, we define staff nurse clinical leaders (SNCL) as registered nurses (RN), with a bachelor degree in nursing, who are directly involved in providing nursing care at the bedside and who exert significant influence on assistant nurses (AN), although no formal authority has been vested in them. The aim of this study was to describe registered nurses' perceptions of what it entails to be the leader at the bedside in inpatient physical care.

## 2. Materials and Method

This study is part of a larger project concerning staff nurses' clinical leadership (SNCL) in nursing care and the perspective of both nurses and head nurses. It is limited to inpatient physical care and is based on interviews with experienced nurses. The study has a descriptive, qualitative, and phenomenographic approach. This is appropriate for mapping qualitatively different ways through which people experience, conceptualise, perceive, and understand various aspects of a phenomenon around them [20]. The goal is to identify and describe the various ways in which people experience the phenomenon [21]. The relative strength of phenomenographic studies lies in their emphasis on variation between peoples' various ways of experiencing and conceiving the world around them [22]. Accordingly, the outcome categories from a phenomenographic analysis constitute peoples' various ways of thinking about their experiences [22]. Marton [20] made a distinction between research undertakings belonging to either the first-order perspective, what something is, or the second-order perspective, how something is perceived to be. The second-order perspective is the essential one in phenomenography. The result is a description on a collective level of the logical relationship between descriptive categories and conceptions [23]. It contributes with structural and content aspects of how phenomena are experienced in different situations.

The selection of nurses was purposeful and the intention was to have interested participants able to contribute with a scope of perceptions. Marton [20] means that motivation to participate in the investigation is crucial. Fifteen registered nurses were recruited from inpatient physical care in three central hospitals in Sweden. They worked on different wards: general surgical, gynaecological, ear, nose, and throat, stomach and intestinal, kidney and dialysis, neurological, heart failure, and rehabilitation wards. The head nurse of the wards was contacted by telephone by the first author (Inga E. Larsson) and given information. All nurses on the wards were sent written information regarding aim and procedure by e-mail. Fifteen nurses agreed to participate. After written informed consent to participate had been obtained, the participant chose where and when to meet for an interview. The participants included three men and twelve women, seven from surgical wards and eight from medical wards. The age range was 22–54 years (mean 42) and they had worked as registered nurses for 2–15 years (mean 7) and had a bachelor degree (13) or a master degree (2). None of the participants had management education.

Collection of data was made by means of open interviews and carried out as a dialogue as recommended by Marton [23]. All interviews were held adjacent to the wards in a place where there would be no interruption in order to provide a relaxed environment. The interviews were conducted in an open and friendly atmosphere by the first author (Inga E. Larsson) and lasted 45–90 minutes. All interviews began with two questions: (1) what does it mean to you to be the leader at the bedside? And (2) what do you do as the leader at the bedside? Follow-up questions used were as follows: What do you mean by…? Can you describe an explicit situation for what you mean? And Can you provide further details? These questions were posed in order to clarify the meaning, deepen understanding of the answers, and ensure that the participant had been correctly understood. Each interview was audiotaped and transcribed verbatim in its entirety by the first author (Inga E. Larsson).

Ethical approval for the study was given by the Ethics Review Board at University West (number 2016:7). The Declaration of Helsinki was adhered to [24]. The participants were informed that participation was voluntary and could be halted at any time without explanation, and they were assured of confidentiality. All participants gave their written consent after receiving verbal and written information.

The phenomenographic analysis focuses on similarities and differences between individual statements and based on this the conceptions are grouped in nonoverlapping descriptive categories [23]. Since descriptive categories relate conceptions to each other, they also relate to statements and the whole text [20]. The analysis followed the phenomenographic approach described by Marton [23]. The data material was read repeatedly as open-mindedly as possible to gain an overall sense of the content. Statements in accordance with the aim of the study were identified. These statements were compared in order to find similarities and differences, identifying distinct expressed ways of understanding or experiencing the phenomena. Each statement was labelled and then grouped together as emerging conceptions. To obtain an overall map of possible links between similarities and differences, the conceptions were compared and grouped into preliminary descriptive categories. The focus now changed from relations between the conceptions to relations between the preliminary descriptive categories. The system of conceptions and descriptive categories was scrutinized and checked against statements and the whole data material. The researchers performed the analysis independently and then compared and discussed the results until consensus was reached. By switching focus between the whole and the parts, five established descriptive categories and 12 conceptions finally emerged (Table 1). The 15 interviews included 354 statements.

## 3. Results

### 3.1. Demonstrating Clinical Knowledge

This descriptive category contains two conceptions: handling clinical duties securely and being underpinned with scientific sources.

#### 3.1.1. Handling Clinical Duties Securely

This conception concerns the fact that nurses deliberately demonstrate secure handling to coworkers in both medical care and how they act in acute situations safeguarding patient safety. Nurses' experience and knowledge of the work in the unit develop over time and lead to greater safety at every stage and also enhance structuring of daily work. With increased knowledge, nurses' authority is strengthened. “*When I demonstrate my knowledge, they see that I'm skilled and confident. I notice that they listen more to what I say. I want to do everything right so that they see that I can manage the situation, that there's order and structure, so they look up to me. Sure, there are things I don't know, but I can find out.*”

#### 3.1.2. Being Underpinned with Scientific Sources

This conception is about nurses making use of scientific sources to justify decisions and measures and to demonstrate relevant knowledge. This is regarded as creating a solid basis and ensuring that nursing measures are carried out correctly and in a way that safeguards patient safety. By searching for regulations on Internet and in steering documents, evidence is also used, for example, regarding safety of personnel when there is a risk of infection. This is regarded as strengthening trust in the nurse's knowledge. In development and quality enhancement of nursing care, research is used as an argument for changes. “*It's difficult to tell an assistant nurse who has worked for 30 years that you're doing it wrong. First, you have to make it clear that she has competence, but that the procedure is different now due to new research. For example, that inserting an indwelling catheter has been replaced by intermittent catheterization and bladder training. This change increased the assistant nurses' workload. Then I had to underpin this changed routine with research, gave repeated information, explained why and discussed patiently.*”

### 3.2. Establishing a Good Atmosphere of Collaboration

This descriptive category comprises three conceptions: mutual respect, courage to be honest, and involving others by encouraging reflection.

#### 3.2.1. Mutual Respect

This conception refers to nurses being sensitive to AN's verbal and nonverbal signals so they feel seen, heard, and valuable. This also includes listening long enough and taking into consideration what they say. Nurses listen to opinions expressed by others but also pay attention to their own feelings, experiences, and ideas. They explain that everybody should show each other respect regardless of the number of years in the profession, age, or where the person was born. Coworkers need to show understanding for each other's work and not just do as they feel fit. “*I don't want to say to somebody: do that and that; instead, everybody's opinions are important and we reach an agreement. If somebody else suggests that we could do it better this way, then I listen and change my mind if I feel that it's better.*”

#### 3.2.2. Courage to Be Honest

This conception is about nurses wanting to have open and frank communication characterised by genuineness so that everybody can present their views. This presupposes that nurses can be themselves and stand for what they say and that words and actions are congruent. The largest challenge facing a leader is to be able to fearlessly give and accept feedback. This necessitates an open atmosphere and that everybody dares and feels free to express their opinion. Nurses need to be aware that what they feel comfortable with could be challenging for an AN. Criticism needs to be a natural part of collaboration and adapted individually. Nurses may sometimes choose to criticise indirectly because they are uncertain as to how the AN will react. This is exemplified by removing the problem from the AN and discussing it on a general level and from the patient perspective. “*I have to say when something isn't good, otherwise the assistant nurse has no chance of improving. Nobody is perfect, everybody can make a mistake and we need to talk about it. If I want to have an open atmosphere, it's important to be able to admit my own mistakes too. When something in the interaction doesn't feel right, I might say: the atmosphere feels weird, how does it feel to you?*”

#### 3.2.3. Involving Others by Encouraging Reflection

This conception concerns nurses asking questions that require reflection instead of automatically just giving an answer. Open questions are used such as what, when, how, where, and who. This, in turn, results in new questions that are regarded as motivating ANs to increase their responsibility for their actions and promoting development of knowledge. Discussions inspire everybody on the team to find alternative solutions: “*In the same way as I mentor nursing students, I ask questions to encourage reflection, for example, based on Gibbs' model of reflection instead of just saying how something should be done. It's also important to show what decisions are based on; you can read that or you'll find it here. Assistant nurses who question are a positive thing, I think. It can lead to discussions that stimulate reflection and contribute to all of us acquiring more knowledge.*”

### 3.3. Consciously Structuring the Work in Order to Ensure Patients' Best Possible Nursing Care

This descriptive category comprises three conceptions: providing coworkers with a complete picture of the patient's situation, assigning and giving specific instructions for the work, and ensuring that patients' needs are met in front of routines.

#### 3.3.1. Providing Coworkers with a Complete Picture of the Patient's Situation

This conception concerns nurses' coordination of the patient's care and giving a well-founded and complete description of their patients to coworkers involved. By means of dialogue, nurses try to find out how patients are feeling, their understanding of the situation, and expectations regarding their own care. Information about the patient is also gathered from coworkers and relatives. The nurse as leader is seen as the spider in the web who coordinates the patient's care by knowing everything. “*I need to have control over everything being planned and having to be done. I must have read everything in patients' records and know everything about my patients and quickly get a clear picture so that I can give this information to everybody on the team; assistant nurses, physiotherapist, counsellor, physician, the patient and relatives.*”

#### 3.3.2. Assigning and Giving Specific Instructions for the Work

This conception deals with nurses' assignment of duties to ANs based on an assessment of their level of knowledge. A shift often begins with the nurse gathering ANs for a brief review of who does what of the duties planned and assignments are usually done in an open dialogue. Nurses sometimes assign duties on the basis of their own plan for the day, including both others and their own work. After the medical ward round, nurse and ANs meet once again for a review of what they will do. The plan is followed up during the day. When ANs' duties involve doing something special with the patient, the nurse explains why and how it should be done so that they understand the context. It is felt that just giving orders does not work. Some ANs need more guidance and others less when they know what to do. Nurses need to be very clear. “*I communicate, analyze and reflect on what works. When I said this, it didn't work. Then I specify in practical terms what has to be done and later I ask: have you done it, otherwise do it now. Sometimes, I remind her in advance and sometimes I write a note for her to fill in and hand back to me, otherwise I know it won't be done.* /…/* It works when everybody knows what they all have to do and we have a continuous dialogue.*”

#### 3.3.3. Ensuring That Patients' Needs Are Met in front of Routines

This conception concerns nurses' planning and evaluation of the nursing care which is built from patient's needs. In nursing documentation, patients' problems and/or needs that have been observed by nurses are noted. Neither routines nor the nurse's or ANs' own wishes are allowed to steer the work; instead, it is the patient's needs, clarified in the nursing documentation. “*Our work must be structured so that everybody has the same goal and do their duties with focus on the patient and not just follow routines. For example, everybody should be washed before breakfast. I might hear from assistant nurses that first the ordering of supplies must be ready. When priority is given to routines and patients' needs come last, you have to tackle this problem and expand understanding by discussing it from different perspectives and talking about the consequences.*”

### 3.4. Customized Presence in the Practical Work with Patients according to Predetermined Prerequisites

This descriptive category contains two conceptions: being approachable to staff and patients and consulted when changes occur in a patient's condition.

#### 3.4.1. Being Approachable to Staff and Patients

This conception concerns nurses showing that they are approachable to ANs and patients. Being visible and “out” with ANs in practical care work is considered to be crucial for the ability to be in control and for monitoring quality of care. Working together strengthens solidarity with ANs as the nurse then understands the work situation. Not being involved too much in practical work with patients and thus neglecting work that only nurses can carry out are a balancing act. “*I can't lead the work if I'm not with my patients. I usually have medicine trolley and computer with me and I'm always sensitive to what is going on around the patients.*”

#### 3.4.2. Being Consulted When Changes Occur in a Patient's Condition

This conception concerns the fact that nurses should immediately be contacted by the ANs when something deviates from what is normal for the patient. Instead of their own observations of patients, nurses often need to rely on second-hand information from ANs. This is a consequence of an overstretched and heavy work situation with too many patients per nurse. The nurse does not have time to be with each patient as long as what is actually necessary. This requires prioritisation of actions that only a nurse has competence and obligation to carry out, such as medical-technical and acute measures. This places demands on being able to organize one's own work efficiently but also on being a problem-solver who can quickly identify other alternatives. “*I can't be everywhere. I have to be told directly if, for example, a patient is suffering from a cold sweat, is pale or says that he isn't feeling well so that I can do something about it. When I have too many patients, I have to reprioritise and do what I'm first and foremost is required to do as a nurse. I then have to see to it and trust that other tasks are carried out by the assistant nurses.*”

### 3.5. Monitoring Coworkers' Professional Practice

This descriptive category comprises two conceptions: keeping an eye on the interaction between assistant nurses and patient and keeping an eye on new staff.

#### 3.5.1. Keeping an Eye on the Interaction between Assistant Nurses and Patient

This conception is about nurses observing ANs' interaction in their encounters with patients. This can be initiated by an AN having behaved unpleasantly towards a patient or complaints from close relatives. It can also be a consequence of AN having talked over the head of a patient and asked the nurse instead of talking directly to the patient. Nurses also observe to make sure that nobody aggravates or exploits the weak position a patient is in. Keeping an eye on interactions can take the form of direct observation of how a task is performed or indirect observation by standing in the corridor outside a room and listening to how an AN treats patients. “*I feel responsible for how assistant nurses behave. When I suspect that an assistant nurse is not actively present and attentive to a patient or doesn't reflect on how a measure feels to him, I tackle the problem. When, for example, we wash a patient together, I look first at how she's doing it and then I think aloud on purpose. I don't tell the assistant nurse how to do her job; instead, I ask the patient: is it OK like this, does it hurt, how can we do to make it feel better. The way I talk affects the care we give together. I lead by trying to influence how we think in interactions with patients in our daily work.*”

#### 3.5.2. Keeping an Eye on New Staff

This conception is about nurses keeping an eye on persons who have just joined the unit so that patients' safety is not endangered as a result of insufficient knowledge. Getting to know and assessing a new AN's level of knowledge can be achieved by performing a task together. The AN is given opportunity to change a wound dressing so that the nurse can be certain that the measure is based on insight, for example, wearing apron and gloves and doing everything correctly. “*I keep an eye on all new assistant nurses and sometimes I have to ask for an explanation of what she's doing. I always make it clear to new staff members that they must ask if there is something they are uncertain about or don't know. Assistant nurses and even registered nurses can be dangerous if they doesn't ask and makes a mistake.*”

## 4. Discussion

This study provided a structure of descriptive categories and conceptions of registered nurses' perceptions of what it entails to be the leader at the bedside in inpatient physical care. The findings confirm and add to previous work on what it entails to be nurse leader at the bedside. However, our findings highlight these nurses' perceptions of approach and procedures and clarify elements and process, which few researchers have elaborated earlier. One explanation may be that most of the research has investigated formal leadership practices such as those of managers and head nurses. Formal leadership models, for example, transformational leadership, have been claimed to be transferable to nurses leading at the bedside. Our participants' leader principles seem to be grounded in the core values of the nursing profession that ensure nursing values and person-centered attributes ahead of those associated with the dominant groups of managers and physicians (cf. [17]). These are foremost loyal to the organization and its ideology of New Public Management and economism, which often conflict with nurses' professional community values and beliefs about patient care [25]. A leader needs to assume responsibility for caring as the subject matter and give direction towards patients' vulnerability and suffering (cf. [26]). Leadership can then be interpreted as empowerment to focus on patients' interests as the guiding principle.

Our findings point to a social process of deliberate effort to attain and maintain trust, leader status, and authority, in line with Chávez and Yoder [1]. Since nurses have an informal leadership role they need to acknowledge their role as leaders both to themselves and to coworkers or somebody else will take the lead. The findings also point to a deep sense of professional responsibility, but it is up to the coworkers to decide whether the leadership is experienced as “responsible leadership” or not. This leadership involves nearness and sensitivity, distance, understanding, and reflection, as noted by Foss et al. [26]. Our findings also indicate that a nurse leader adheres to the same core values in relation to both patients and coworkers.

*Demonstrating clinical knowledge* points to necessity of attaining status by achieving authority as a leader. As nurses have an informal leadership position, trust in them is essential. This is in line with Stanley [19] who claims that ANs gain confidence in nurses as leaders when they have skills to demonstrate clinical knowledge such as knowledge of nursing and knowledge linked to the specific area of practice. The ability to lead is based on a solid and up-to-date understanding of the basis of professional nursing, in line with Sahlsten et al. [27]. Understanding forms how nurses develop their professional autonomy, which can lead to different ways of thinking. Accordingly, nurses in the unit need theoretical knowledge to discuss and reach a consensus on the meaning of professional responsibility to lead at the bedside.

*Establishing a good atmosphere of collaboration* clarifies the significance of a dialogue and the leader's responsibility for creating a good climate of honesty and authenticity that makes it possible to build confidence in the nurse as a role model. This is recognized as one of the elements in congruent leadership [17]. If the nurse and AN have confidence in each other, it is easier to be honest and they can demand more of each other. In a secure but also challenging collaboration, the nurse can test new ideas and way of leading in order to develop courage and knowledge of both parties. A prerequisite is to reflect together in order to utilise the AN's potential for providing care. This can be traced to “reflection in action,” a conscious reflection during the course of the action, followed by “reflection on action” after the action in order to raise awareness of reflected knowledge [28]. As leader, a nurse also needs to be perceptive and confirming so that each individual coworker feels valuable and appreciated and is able to develop in his/her work. This is in line with Bondas [29] who claims that a trustful atmosphere requires human love visible in mutual response, tolerance, and respect for the individual.

*Consciously structuring the work in order to ensure patients' best possible nursing care* places high demands on the nurse as leader as it involves being an effective coordinator and communicator and assumes the responsibility to be in control. Being an effective communicator requires excellent listening skills and ability to clearly articulate and share clinical information, observations, and opinions, also pointed to by Stanley [19]. Our participants display values and beliefs through their actions and stimulate ANs to see and deepen their understanding of clinical situations from different perspectives. Sometimes, instead of verbal criticism, a nurse makes indirect remarks to stimulate reflection. Congruence with professional values, empathy, and nonjudgementalism are qualities Rogers [30] identified as prerequisites for leading, teaching, and counselling, activities involved when a nurse leads coworkers.

*Customized presence in the practical work with patients according to predetermined prerequisites* shows that some participants can be available to coworkers and patients during the day and others are consulted in the event of changes in the patient's condition. This clarifies that nurses adapt their way of leading in accordance with the prerequisites a nurse has to relate to here and now. This is in line with situational leadership theory based on formal leadership practices [31]. Even though none of the participants mentions any theoretical basis for their leadership, this model can still be identified. They seem to act as participating leaders, a low task and high relationship style that passes on day-to-day decisions such as task allocation to the followers (cf. [31]). Why nurses do not mention any theoretical model for leadership may depend on lack of clarity regarding their leader position as nurses both during their nursing education and from their organization. Registered nurses may seldom been recognized as the leader at the bedside in hospitals. Accordingly, nurses maybe do not see themselves as the leader. Another explanation may be that research has not focused on nurses' informal leader position.

To lead the nursing care is a question of organizing one's own and ANs' work while adapting to each person's competence level and ability to take responsibility. A nurse needs to learn about and become involved in the AN's continued development. The main responsibility for allocating and organizing resources and for development of coworkers' competence lies with the first-line manager of the unit. However, everybody has a responsibility, which means that coworkers need to be reminded that they are part of the work environment. Previous research has shown that both nurses [27] and patients [32, 33] emphasise the importance of a person-centered organization where patients have their own nurse during their hospital stay. When nurses have too many patients, they are dependent on second-hand information from ANs instead of being present and quickly initiate actions. Aiken et al. [34] have shown that too many patients per nurse increases mortality, number of infections, and medical injuries. This causes suffering and unnecessary costs, which could instead be used to increase the number of nurses.

*Monitoring coworkers' professional practice *does not seem to have emerged so clearly in previous studies of nurses' leadership at the bedside. Here, nurses assume leadership accountability for patient safety in order to protect them from violations and abuse of power or insufficient knowledge that could pose a risk. If patient's integrity and autonomy are not respected, the nurse is obligated in her capacity as leader to deal with the problem [35]. This is also in line with Bondas [29] who states that there is no tolerance for violations of human dignity. Patients are the group with least power and influence and are at the bottom of the power hierarchy in the health care sector [33]. Our results show that nurses monitor the interaction between ANs and patients so that the asymmetry is not exploited or reinforced. The nurse is a key person and which kind of leadership he/she performs is crucial for the situation whether a patient is guaranteed legal right to participate in their own care or not [36]. Accordingly, the patient's perspective on their own situation constitutes the basis and core of leading person-centered nursing care.

Our findings are based on these participants (*n* = 15) and their ability to describe conceptions and experiences. The descriptions are related to a group level rather than to the individual participants. The findings describe the qualitatively different ways the participants experience the situation. When 13 interviews had been conducted, earlier data were replicated and nothing new was added. The participants contributed a broad, rich, and detailed variation of experience-based data. Two researchers independently performed an analysis and compared structures of descriptive categories and conceptions. According to Marton [23], there should be at least a two-thirds agreement when comparing analyses of two independent and competent researchers and in our case consensus was reached. The credibility of the findings mainly concerns the relationships among the categories and the data and is thus strengthened by direct quotations (cf. [23]).

The findings of this study should be interpreted in light of a few limitations. First, this study is limited to physical inpatient care and the reader has to decide if the findings are transferable to their own context. Obviously, other nurses and settings need to be explored. Second, several factors may influence a nurse in being a leader and studies are lacking of hindrances/barriers nurses experience as well as what promotes their leadership. Third, as nurses adapt their way of leading in accordance with the prerequisites they have to relate to, nurse staffing levels influences need to be investigated. Fourth, the perspectives of head nurses, ANs, and physicians are lacking in this study and would be essential to obtain in order to develop an in-depth understanding of issues surrounding being a leader at the bedside.

## 5. Conclusions

This study provided a structure of being a leader at the bedside in inpatient physical care based on Swedish registered nurses' perceptions. Leader principles grounded in the core values of the nursing profession that ensure nursing values and person-centered attributes were a key aspect. Our results highlight a social process of deliberate effort to attain and maintain leader status and authority. Deliberate communicative approaches and interactive procedures were identified as five descriptive categories: demonstrating clinical knowledge, establishing a good atmosphere of collaboration, consciously structuring the work in order to ensure patients' best possible nursing care, customized presence in the practical work with patients according to predetermined prerequisites, and monitoring coworkers' professional practice. As the leadership role is influenced by predetermined prerequisites in the context, further studies are essential.

The findings from this study are essential in teaching nurse students what it means to lead at the bedside. It can also provide guidance in drafting of a job description for SNCL. Accordingly, the findings can contribute to a common understanding of the phenomenon among nurses and differentiate it from leading in general whatever professional area, to lead as a head nurse, and, as such, form a basis for discussions and further research.

## Figures and Tables

**Table 1:** The results describe registered nurses' perceptions of what it entails to be the leader at the bedside in inpatient physical care.

Descriptive categories	Conceptions
Demonstrate clinical knowledge	Handling clinical duties securely
	Being underpinned with scientific sources

Establish a good atmosphere of collaboration	Mutual respect
	Courage to be honest
	Involving others by encouraging reflection

Consciously structuring the work in order to ensure patients' best possible nursing care	Provide coworkers with a complete picture of the patient's situation
	Assign and give specific instructions for the work
	Ensure that patients' needs are met in front of routines

Customized presence in the practical work with patients according to predetermined prerequisites	Be approachable to staff and patients
	Consulted when changes occur in a patient's condition

Monitor coworkers' professional practice	Keeping an eye on the interaction between assistant nurses and patient
	Keeping an eye on new staff
